# Supplementary material for: From sequence to enzyme mechanism using multi-label machine learning
Source: BMC Bioinformatics. 2014 May 19;15:150. doi: 10.1186/1471-2105-15-150 (PMC4229970; doi:10.1186/1471-2105-15-150)
Supplement: Additional file 2 — Java code of ml2db. Additional file ml2db_code.tar.gz contains the Java source code to run the multi-label machine learning experiments and save the results to database. The code’s Javadoc is included. [file 1471-2105-15-150-S2.zip › additional file 2/ml2db/ml2db/doc/mulan/classifier/transformation/package-summary.html]

mulan.classifier.transformation


JavaScript is disabled on your browser.


- Overview
- Package
- Class
- Use
- Tree
- Deprecated
- Index
- Help

- Prev Package
- Next Package

- Frames
- No Frames

- All Classes

# Package mulan.classifier.transformation

- Class Summary

  | Class | Description |
  | --- | --- |
  |  |  |
  | --- | --- |
  | AdaBoostMH | Implementation of the AdaBoost.MH algorithm based on Weka's AdaBoostM1. |
  | BinaryRelevance |  |
  | CalibratedLabelRanking | Class implementing the Calibrated Label Ranking (CLR) algorithm. |
  | ClassifierChain | Class implementing the Classifier Chain (CC) algorithm.    For more information, see    Read, Jesse, Pfahringer, Bernhard, Holmes, Geoff, Frank, Eibe: Classifier Chains for Multi-label Classification. |
  | EnsembleOfClassifierChains | Class implementing the Ensemble of Classifier Chains(ECC) algorithm. |
  | EnsembleOfPrunedSets | Class implementing the Ensemble of Pruned Sets algorithm(EPS) . |
  | IncludeLabelsClassifier | A multilabel classifier based on the include labels transformation. |
  | LabelPowerset | Class that implements a label powerset classifier |
  | LabelsetPruning | Common functionality class for the PPT and PS algorithms |
  | MultiClassLearner |  |
  | MultiLabelStacking | This class is an implementation of the (BR)^2 or Multi-Label stacking method.    For more information, see    Grigorios Tsoumakas, Anastasios Dimou, Eleftherios Spyromitros, Vasileios Mezaris, Ioannis Kompatsiaris, Ioannis Vlahavas: Correlation-Based Pruning of Stacked Binary Relevance Models for Multi-Label Learning. |
  | PPT | Class implementing the Pruned Problem Transformation algorithm (PPT) . |
  | PrunedSets | Class implementing the Pruned Sets algorithm (PS). |
  | TransformationBasedMultiLabelLearner |  |
- Enum Summary

  | Enum | Description |
  | --- | --- |
  |  |  |
  | --- | --- |
  | PPT.Strategy | strategies for processing infrequent labelsets |
  | PrunedSets.Strategy | strategies for processing infrequent labelsets |

- Overview
- Package
- Class
- Use
- Tree
- Deprecated
- Index
- Help

- Prev Package
- Next Package

- Frames
- No Frames

- All Classes
